# Comparison of the Efficacy Between Ultrasound-Guided Paravertebral Block and Erector Spinae Block for Postoperative Analgesia in Percutaneous Nephrolithotomy Using Levobupivacaine: A Prospective and Randomized Study

**DOI:** 10.7759/cureus.67401

**Published:** 2024-08-21

**Authors:** Karthik GS, Sudheer Ramegowda, Mahesh Chandra, Ashwani Kristipati, Prajyot Bhurli, Alieshia Siangshai

**Affiliations:** 1 Anaesthesiology and Critical Care, Rajarajeshwari Medical College and Hospital, Bangalore, IND; 2 Anaesthesiology, Rajarajeswari Medical College and Hospital, Bangalore, IND

**Keywords:** percutaneous nephrolithotomy (pcnl), ultrasound (u/s), paravertebral block (pvb), levobupivacaine, erector spinae plane block (espb)

## Abstract

Introduction

Various techniques have been developed in the current era of regional anesthesia practice. With the advent of ultrasound, the visualization of needle and pleura in real time enables a better outcome with negligible adverse events. This study was designed to compare the efficacy between ultrasound-guided erector spinae plane block (ESPB) and paravertebral block (PVB) in percutaneous nephrolithotomy (PCNL) for the duration of postoperative analgesia with levobupivacaine, a local anesthetic with higher lipid solubility, making it more potent and resulting in a longer duration of action.

Methods

This prospective randomized single-blinded study enrolled 50 patients of ASA grades I and II, aged between 20 and 60 years, who were scheduled for PCNL under general anesthesia. Patients were divided into two groups of 25 each: group ESPB and group PVB, and 25 mL of 0.25% levobupivacaine was administered to both groups. They were primarily evaluated for the duration of postoperative analgesia. Total rescue analgesic requirements, hemodynamic parameters, and any adverse effects were also assessed.

Results

Both ESPB and PVB provided a significant duration of analgesia postoperatively. Demographic characteristics in both groups were comparable. The duration of postoperative analgesia in group ESPB was 746 ± 58.6 minutes when compared to group PVB, which is 768 ± 68.6 minutes (p = 0.08). Intravenous (IV) paracetamol was used as a rescue analgesic. The doses used were also comparable in both groups, with the visual analog score (VAS) being high after around 12 hours of surgery. The total rescue analgesic requirement was similar in both groups (group ESPB, 2.0 ± 1.6; group PVB, 2.2 ± 1.4; p = 0.51). There were no significant hemodynamic or other adverse effects in either group.

Conclusion

We conclude that both ESPB and PVB using isobaric levobupivacaine 0.25% as a local anesthetic are equally efficacious in providing effective postoperative analgesia in patients undergoing PCNL under general anesthesia.

## Introduction

Percutaneous nephrolithotomy (PCNL) is a minimally invasive gold-standard surgical technique for managing complicated renal stones compared to open surgery [1,2]. Pain after PCNL is caused by the distension of the renal capsule, parenchymal tract, skin incision, subcutaneous tissue, and muscles [3]. Various analgesic techniques have been advocated as adjuncts to general anesthesia in many surgeries [1,4]. Regional anesthesia plays a crucial role in enhancing patient care, improving the quality of treatment, and increasing patient satisfaction. Ultrasound-guided visualization of the needle in fascial planes ensures optimal positioning, thereby preventing injury to adjacent anatomical structures [5,6].

Erector spinae plane block (ESPB) is an interfascial blockade, first described by Forero et al. in 2016, which involves the injection of a local anesthetic into a fascial plane placed between erector spinae muscles and the tip of the transverse vertebral process [7]. This targets the origin of spinal nerves, as demonstrated in various cadaveric and contrast studies, providing analgesia in different clinical scenarios with a wide spectrum of applications [8,9]. Paravertebral block (PVB) injects local anesthetic adjacent to the vertebra where the spinal nerves emerge from the intervertebral foramina. The paravertebral space accommodates local anesthetic, which can spread into cephalad, caudal, intercostal, intrapleural, epidural, and prevertebral spaces [10,11]. Though this technique is not devoid of adverse effects, it can be minimized with proper anatomical knowledge and the use of ultrasound [12]. This results in nerve blockade for multiple contiguous thoracic dermatomes above and below the site of injection, and it is effective in treating acute and chronic pain of unilateral origin from the chest and abdomen [13].

Levobupivacaine has similar pharmacodynamics as bupivacaine, but the decreased cardiovascular and central nervous system toxicity profile makes it a preferred drug of choice with a greater margin of safety. Its higher lipid solubility makes it more potent, resulting in a longer duration of action [14,15]. Extensive research has evaluated the use of local anesthetics in various fascial plane blocks. However, studies employing 0.25% isobaric levobupivacaine have not been substantially studied, and there is a lack of comprehensive works of literature using levobupivacaine as an adjunct to general anesthesia in patients undergoing PCNL.

Hence, we designed this study to compare the efficacy between ESPB and PVB using 0.25% levobupivacaine under ultrasound guidance in PCNL surgeries.

## Materials and methods

This prospective randomized study was conducted in the Department of Anesthesia, Rajarajeswari Medical College and Hospital, Bengaluru, India, for a period of six months from August 2023 to January 2024. The study was performed to compare the analgesic efficacy between ultrasound-guided ESPB and PVB in PCNL under general anesthesia using isobaric levobupivacaine 0.25%, besides assessing hemodynamic parameters and other adverse effects.

After obtaining the Institutional Ethical Committee approval (approval letter number: IEC/37/2023 dated May 22, 2023), the study was registered on the Clinical Trials Registry of India (CTRI/2023/ 07/055193). It was conducted according to the guidelines in the Declaration of Helsinki (2013).

Inclusion and exclusion criteria

Fifty patients of ASA grades I and II, aged between 20 and 60 years, scheduled for PCNL surgeries under general anesthesia were enrolled. Patients were excluded from the study in case of block failures, known hypersensitivity to local anesthetics, bleeding disorder, uncontrolled diabetes mellitus, severe renal and liver diseases, epilepsy, mental instability, and refusal to give consent.

Preoperative preparation

Informed written consent was obtained from all the patients willing to undergo surgery. Baseline demographic data included were age, gender, height, weight, and duration of surgery. All patients were subjected to detailed pre-anesthetic workup and evaluation. A day before surgery, patients were re-evaluated, the procedure was explained to them, and they were taught to interpret the visual analog scale (VAS) to assess the duration of postoperative analgesia. Fasting guidelines for solids were maintained for six hours. Patients were pre-medicated with oral alprazolam 0.25 mg and ranitidine 150 mg the night before surgery, and intravenous (IV) ondansetron 4 mg was administered 30 minutes before surgery.

**Figure 1:**
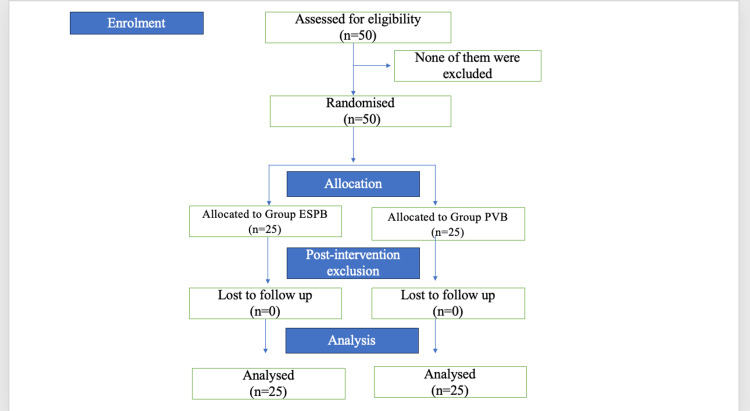
CONSORT flowchart: participants screening and enrolment process CONSORT, Consolidated Standards of Reporting Trials

The study was single-blinded, and patients were randomized and allocated into two groups based on an open-envelope method (Figure 1). Group ESPB and group PVB patients received 25 mL of 0.25% levobupivacaine at the T8 level.

Intraoperative phase

On arrival of the patient to the operation theater, ASA standard monitors were connected, and baseline pulse rate, non-invasive blood pressure (NIBP), oxygen saturation by pulse oximeter, and electrocardiogram (ECG) were recorded. An additional wide-bore 18G cannula was secured, and intravenous fluids were given as required.

Following preoxygenation, as per the standard protocol, general anesthesia was induced with a bolus dose of IV fentanyl (2-2.5 mcg/kg) and IV propofol (1-2 mg/kg) titrated to loss of verbal response. After obtaining adequate muscle relaxation with IV vecuronium 0.1 mg/kg, endotracheal intubation was done. Anesthesia was maintained with a balanced mixture of oxygen, air, 0.8-1 minimum alveolar concentration (MAC) of isoflurane, and intermittent doses of IV vecuronium (0.01-0.02 mg/kg).

Patients were put in a lithotomy position to facilitate ureteral catheterization by urologists. Patients were then turned to a prone position for PCNL, and hemodynamic parameters were monitored throughout the procedure at regular intervals. After the completion of the surgery before extubation, the study blocks, as narrated above, were administered to the patients as per the allocation under ultrasound guidance (using the M-turbo USG system by Fujifilm Sonosite, Inc., Bothell, WA, USA). Under strict aseptic conditions, counting down from the spine of the seventh cervical vertebrae and under ultrasound guidance, the eighth thoracic vertebrae (T8) was identified. A curvilinear ultrasound probe was placed across the T8 transverse process and moved to a vertical alignment, and the erector spinae muscle was visualized using an in-plane technique. An 18G Tuohy’s needle attached to a 10 cm extension was advanced toward the transverse process underneath the anterior fascia of the erector spinae muscle, and 3-5 mL of normal saline was injected to detect adequate placement. After confirmation of spread, 25 mL of 0.25% levobupivacaine was injected.

Similarly, in group PVB, the transverse process at the T8 level was visualized, and cautious hydrodissection was done using around 4-5 mL of normal saline to reach the paravertebral space. This prevented pleural injury and facilitated adequate placement of the drug. The drug (25 mL of isobaric levobupivacaine 0.25%) was deposited in the paravertebral space between the costotransverse ligament and pleura (which was visualized as a hypoechoic layer). The patients were shifted to a supine position and extubated after the reversal of residual neuromuscular block with IV neostigmine (0.05 mg/kg) and IV glycopyrrolate (0.01 mg/kg). Hypotension was considered if the decline in blood pressure was 20% below the baseline. This was treated with IV ephedrine 6 mg boluses. Bradycardia was defined as a heart rate of less than 50 beats/minute, which was managed with IV atropine 0.6 mg bolus. Postoperative nausea and vomiting were tackled with IV ondansetron 4 mg.

Postoperative phase

Patients were transferred to the post-anesthesia care unit. They were evaluated for the duration of postoperative analgesia, and pain was assessed using a standard 10 cm linear VAS. Pain assessment was done 20 minutes after extubation, which was considered zero time. When the VAS score was more than or equal to 4, IV paracetamol 1 g was given as a rescue analgesic, and the total number of doses of IV paracetamol 1 g given in 24 hours was computed. The total duration of analgesia was taken from zero time till the first rescue analgesic requirement. Hemodynamic parameters and other adverse effects and postoperative pain at zero, one, two, four, six, eight, 12, 16, 20, and 24 hours.

Sample size calculation and statistical analysis

As per the study conducted by Lomate et al. [2], having a 5% alpha level of significance and 90% power of the test to detect a difference of 9.2 hours between two groups for the duration of analgesia and considering the 10% possible dropouts, 25 patients per group were included in this study.

The collected data was tabulated using the IBM SPSS Statistics, version 26.0 (IBM Corp., Armonk, NY). Mean and standard deviation were calculated for continuous variables and VAS scores. Categorical data was represented in the form of frequencies and proportions. The unpaired T-test and chi-square test were applied to compare two independent groups. The Mann-Whitney U test was applied to compare two independent ordinal variables. The normality of the continuous data, if compared, was tested by the Kolmogorov-Smirnov test and the Shapiro-Wilk test.

A p-value of <0.05 was considered statistically significant, and a value less than 0.001 was highly significant.

## Results

The total number of patients recruited for the study was 50 patients divided into two groups. None of them were excluded from the study. Both groups were comparable in terms of age, weight, height, sex distributions, ASA grading, and duration of surgery (p > 0.05) (Table 1 ).

**Table 1:** Demographic characteristics and duration of surgery in the study groups Demographic characteristics were comparable. Unpaired T-test was used; p > 0.05, not significant; p < 0.05, significant; p < 0.001, highly significant. The chi-square test was used for ASA and gender distribution; p < 0.05, statistically significant. kg, kilograms; cm, centimeters; min, minutes

Variable	Group ESPB	Group PVB	Unpaired-T test value	p-value
Age (years)	35.6 ± 6.1	36.1 ± 7.2	0.3747	0.7
Weight (kg)	55.6 ± 4.7	56.9 ± 3.7	1.536	0.12
Height (cm)	161.9 ± 5.92	162.2 ± 3.2	0.31'5	0.75
Duration of surgery (min)	97 ± 4.16	98.2 ± 3.4	1.579	0.11
Variable	Group ESPB	Group PVB	p-value (chi-square test)	
ASA				
Grade 1	13 (52%)	12 (48%)	0.77	
Grade 11	12 (48%)	13 (52%)		
Gender				
Male	10 (40%)	13 (52%)	0.57	
Female	15 (60%)	12 (48%)		

Both groups were successfully given USG-guided ESPB and PVB, respectively. According to our observations, the duration of analgesia was equally efficacious in both groups.

The duration of postoperative analgesia in group ESPB was 746 ± 58.6 minutes when compared to group PVB, which is 768 ± 68.6 minutes (p = 0.08), while the total analgesic requirement in group ESPB was 2.0 ± 1.6 g when compared to group PVB, which is 2.2 ± 1.4 g (p = 0.51). Hence, this was comparable among both groups. IV paracetamol was used as a rescue analgesic. The doses of paracetamol used were also comparable in both groups, with VAS being high in both groups after around 12 hours of surgery (Table 2). Both groups had a superior analgesia efficacy up to around 12 hours, exhibiting statistical significance.

**Table 2:** Comparison of duration of analgesia, total rescue analgesia requirement, and VAS scores postoperatively in both groups The duration of postoperative analgesia in group ESPB vs. PVB was 746 ± 58.6 vs. 768 ± 68.6 minutes, respectively, with p = 0.08, while the total analgesic requirement in group ESPB was 2.0 ± 1.6 g when compared to group PVB, which is 2.2 ± 1.4 g (p = 0.51). IV paracetamol was used as a rescue analgesic. The doses of paracetamol used were also comparable in both groups, with the VAS being high in both groups after around 12 hours of surgery. The p-values are obtained using the Mann-Whitney U test; p < 0.05, statistically significant; p > 0.05, not statistically significant. hrs, hours; min, minutes; g, grams; VAS, visual analog scale

Variable	Group ESPB	Group PVB	p-value
Duration of postoperative analgesia (from zero time in min)	746 ± 58.6	768 ± 68.6	0.08
Total rescue analgesic requirement (paracetamol given in g)	2.0 ± 1.6	2.2 ± 1.4	0.51
VAS score (post-op) (hrs)			
0	2.2 ± 0.62	2.1 ± 0.86	0.5
1	2.8 ± 0.96	2.6 ± 0.42	0.18
2	2.2 ± 0.34	2.3 ± 0.48	0.23
4	1.86 ± 0.62	1.78 ± 0.70	0.5
6	1.98 ± 0.16	1.96 ± 0.38	0.73
8	2.06 ± 0.4	2.10 ± 0.62	0.7
12	7.72 ± 0.32	7.60 ± 0.8	0.35
16	6.8 ± 0.58	6.7 ± 1.12	0.57
20	6.4 ± 0.46	6.5 ± 0.92	0.49
24	6.20 ± 1.34	6.30 ± 1.58	0.73

**Figure 2:**
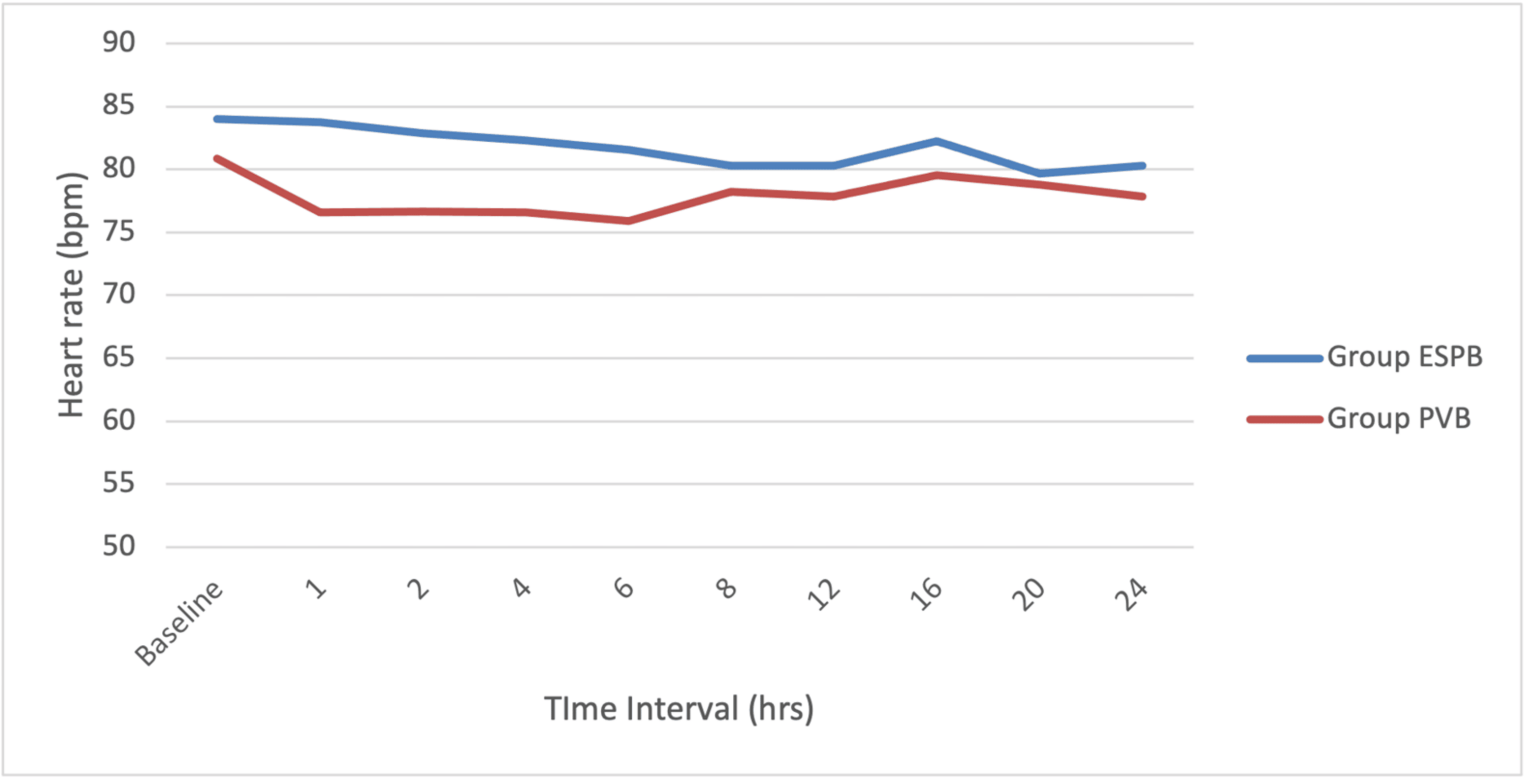
Line diagram showing heart rate distribution at various time intervals among the study participants ESPB, erector spinae plane block; PVB, paravertebral block; hrs, hours; bpm, beats per minute

**Figure 3:**
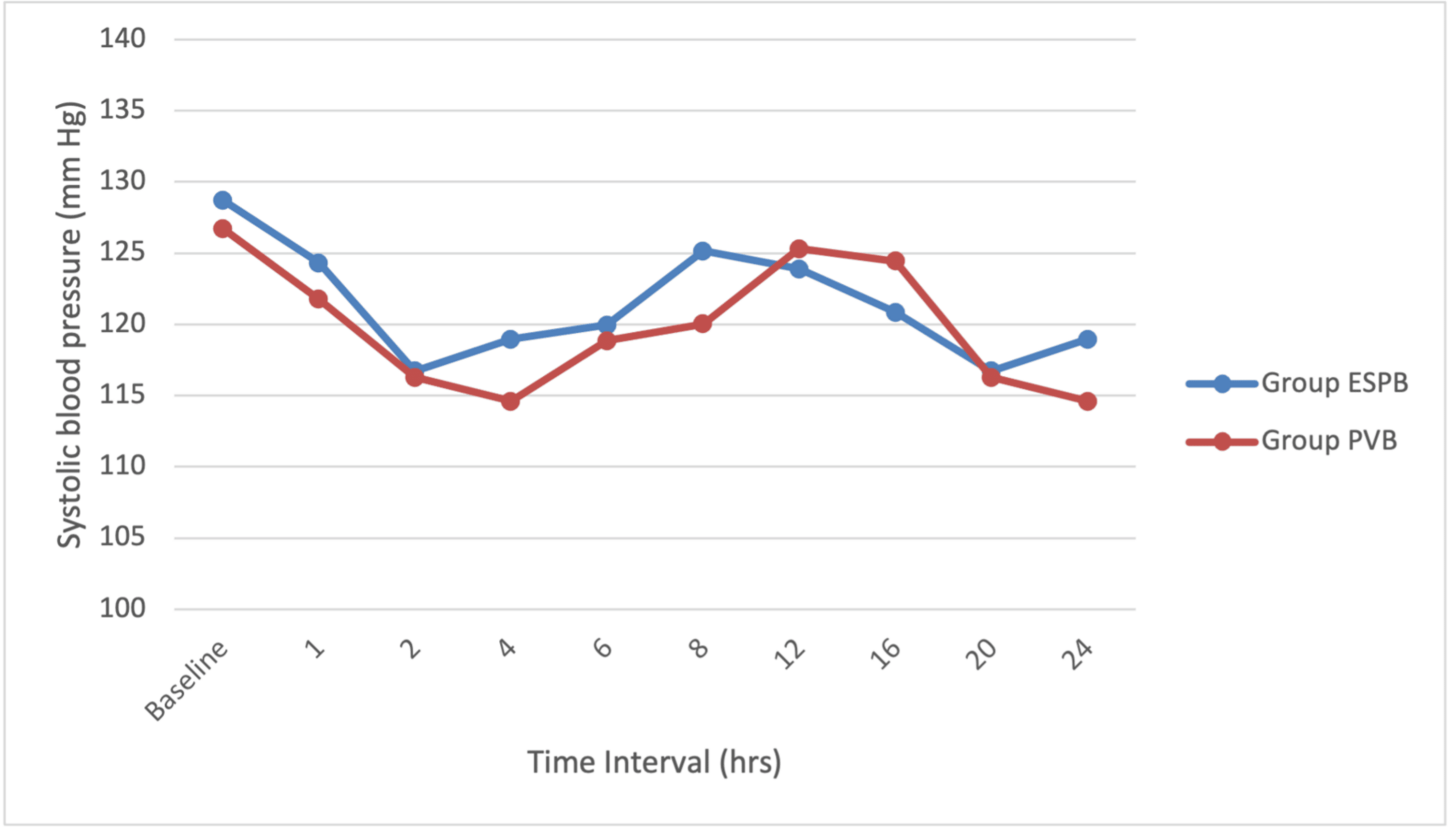
Line diagram showing systolic blood pressure distribution at various time intervals among the study participants ESPB, erector spinae plane block; PVB, paravertebral block; hrs, hours; mmHg, millimeters of mercury

**Figure 4:**
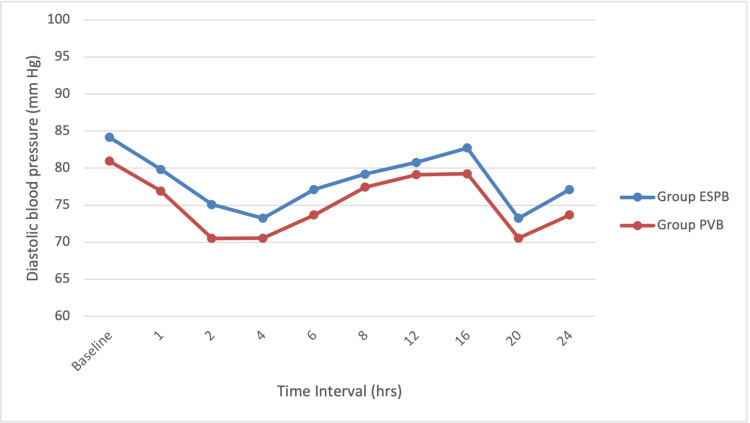
Line diagram showing diastolic blood pressure distribution at various time intervals among the study participants ESPB, erector spinae plane block; PVB, paravertebral block; hrs, hours; mmHg, millimeters of mercury

The mean heart rate and systolic and diastolic blood pressures were comparable intraoperatively and postoperatively in both groups (Figures 2-4). There was no apparent local toxicity in either group. We did not encounter any significant hemodynamic disturbances in either group. None of the patients from either group had adverse effects like nausea, vomiting, and respiratory depression.

## Discussion

Recent advances in regional analgesic techniques have been an increasing trend over recent years for substantial pain relief in numerous surgical procedures. The ESPB is a truncal facial plane block that provides both somatic and visceral analgesia for various surgeries. Local anesthetic is administered in the fascial plane beneath the erector spinae muscle [1,2]. The important concern with the thoracic PVB is its close anatomical proximity to the pleura and central neuraxial system. After the advent of ultrasound, the risk of complications has decreased significantly. Hence, it is also considered one of the efficacious techniques to provide postoperative analgesia with a high success rate [10,11].

The current study was conducted to compare the efficacy between the ESPB and PVB in PCNL. We observed that both the blocks were comparable in providing a similar duration of analgesia. The use of ultrasound prevents injury of the main vessels and pleura [5]. It is also used as a part of multimodal analgesia for enhanced recovery after surgery [6]. Both groups were comparable in terms of age, weight, height, gender distributions, ASA grading, and duration of surgery (p > 0.05). Our study observed that the duration of analgesia in group ESPB was 746 ± 58.6 minutes when compared to group PVB, which is 768 ± 68.6 minutes (p = 0.08). After around 12 hours of surgery, the VAS score was high in both groups (p = 0.35). The doses of paracetamol used were comparable in both groups. The total analgesic requirement was also comparable in these groups (group ESPB, 2.0 ± 1.6 g; group PVB, 2.2 ± 1.4 g), with p = 0.51. IV paracetamol was used as rescue analgesic. We did not encounter any significant hemodynamic disturbances in either group, and none of the patients in either group had any adverse effects.

Our observations corroborated with a similar study conducted by Khot et al. [16]. They compared these blocks using bupivacaine 0.25% before the surgical incision, and patients were monitored intraoperatively and postoperatively for analgesic requirements. They concluded that both blocks were equally efficacious in providing superior analgesic characteristics, which was our primary objective. We preferred levobupivacaine as various studies have well established its safety profile without any adverse cardiac or neurological effects [14]. No studies are available to date regarding the use of these fascial plane blocks just before extubation. Our study was unique and novel as we administered the blocks just before extubation to provide a prolonged duration of postoperative analgesia. Our study also correlated with Elewa et al. [17] to some extent, which was conducted in patients undergoing breast surgeries. In their study, though patients in the erector spinae group had lower morphine consumption than the paravertebral group, patients’ request for the first rescue analgesic in both the groups was after a significantly prolonged time (7.9 ± 1.2 vs. 7.5 ± 0.9 vs. 2 ± 1.2 h; p < 0.001).

Many studies have assessed the effects of ESPB and PVB in various surgeries. Bryniarski et al. [18] performed a clinical trial using an ESPB for postoperative analgesia in PCNL. They evaluated the efficacy and safety of ESPB and observed that the VAS scores postoperatively were significantly lower in the ESPB group (p = 0.01). The need for rescue analgesia was reduced in patients who received ESPB, and thus, they concluded that ESPB provides effective analgesia in the postoperative setting.

Coveney et al. [19] conducted a study to assess the safety and efficacy of the PVB for the operative treatment of breast cancer. They analyzed the parameters, including postoperative pain, nausea, vomiting, and length of hospital stay, in these patients. They postulated that PVB reduced narcotic requirements and provided effective postoperative sensory blockade when compared to general anesthesia alone. A trial conducted by Swisher et al. [20] revealed the superior postoperative analgesic effect of PVB over ESPB in women undergoing nonbreast lumpectomies. They commented about the inferior analgesic efficacy of ESPB over the PVB in terms of opioid analgesic consumption and NRS scale. This study does not comply with our observations.

During the intraoperative period, none of the patients had hemodynamic disturbances. Since both the blocks were done under ultrasound guidance, none of the patients had desaturation, respiratory depression, or pneumothorax. PVBs are technically challenging when compared to ESPB.

Our present study has divulged that both paravertebral and ESPBs provide superior postoperative analgesia. Despite the levels of uncertainty in a few published works of literature, we postulate that both blocks have effective analgesia for patients undergoing PCNL surgeries under general anesthesia.

Limitations of study

This trial was single-blinded and did not involve a large sample size to obtain clinically and statistically valid observations. Further, multiple randomized clinical trials comparing these different regional techniques are needed to validate the feasibility of using these techniques. Only ASA I and II grade patients were involved; henceforth, we did not establish the utility of these techniques in patients belonging to higher ASA grades, which involve moribund patients. We used a single injection to perform the nerve block rather than a catheter, where its utilization would have resulted in a higher patient satisfaction rate, facilitating early ambulation and quicker recovery within the hospital stay. Variable cephalocaudal spread of injectate in these blocks has also been debated. We need to perform a higher number of ultrasound-guided ESPB and PVB to concur with our findings. The feasibility of these techniques needs further evaluation of high-risk and complicated surgeries. We could not assess the overall patient satisfaction score. A multicenter study with a large sample size might yield better outcomes.

## Conclusions

We recommend the widespread use of both these techniques in PCNL. Though PVB has a long and steep learning curve when compared to ESPB, both blocks can be safely used under ultrasound guidance. Levobupivacaine has provided an acceptable analgesic profile in both groups. These techniques are a good supplement to general anesthesia, which is the preferred modality for most intra-abdominal surgeries. Opioid consumption was also reduced in both groups. After the discovery of ultrasound, the reluctance to use these blocks owing to technical difficulties has reduced, and the use of regional anesthetic techniques in our armamentarium has revolutionized. Our study also disclosed that hemodynamic and other adverse complications were negligible. The current study found that both blocks were equally efficacious in providing significant duration of postoperative analgesia and hemodynamic stability.

## References

[1] Ibrahim M, Elnabtity AM (2019). Analgesic efficacy of erector spinae plane block in percutaneous nephrolithotomy: a randomized controlled trial. Anaesthesist.

[2] Lomate P, Jadhav VR, Yadav A (2021). Comparison of the efficacy of erector spinae plane block and peritubal infiltration of levobupivacaine for postoperative analgesia following percutaneous nephrolithotomy. J Anaesthesiol Clin Pharmacol.

[3] Ramachandran S, Ramaraj KP, Velayudhan S, Shanmugam B, Kuppusamy S, Lazarus SP (2021). Comparison of erector spinae plane block and local anaesthetic infiltration of the incision site for postoperative analgesia in percutaneous nephrolithotomy - a randomised parallel-group study. Indian J Anaesth.

[4] Wu H, Ding T, Yan S, Huang Z, Zhang H (2022). Risk factors for moderate-to-severe postoperative pain after percutaneous nephrolithotomy: a retrospective cohort study. Sci Rep.

[5] Kurdi MS, Agrawal P, Thakkar P, Arora D, Barde SM, Eswaran K (2023). Recent advancements in regional anaesthesia. Indian J Anaesth.

[6] Franco CD, Sala-Blanch X (2019). Functional anatomy of the nerve and optimal placement of the needle for successful (and) safe nerve blocks. Curr Opin Anaesthesiol.

[7] Forero M, Adhikary SD, Lopez H, Tsui C, Chin KJ (2016). The erector spinae plane block: a novel analgesic technique in thoracic neuropathic pain. Reg Anesth Pain Med.

[8] De Cassai A, Bonvicini D, Correale C, Sandei L, Tulgar S, Tonetti T (2019). Erector spinae plane block: a systematic qualitative review. Minerva Anestesiol.

[9] Kot P, Rodriguez P, Granell M (2019). The erector spinae plane block: a narrative review. Korean J Anesthesiol.

[10] Gilbert J, Hultman J (1989). Thoracic paravertebral block: a method of pain control. Acta Anaesthesiol Scand.

[11] Eason MJ, Wyatt R (1979). Paravertebral thoracic block-a reappraisal. Anaesthesia.

[12] Hamed IG, Fawaz AA, Rabie AH, Abdallah AEA, Ashoor TM (2020). Ultrasound-guided thoracic paravertebral block vs pectoral nerve block for postoperative analgesia after modified radical mastectomy. Ain-Shams J Anesthesiol.

[13] Ardon AE, Lee J, Franco CD, Riutort KT, Greengrass RA (2020). Paravertebral block: anatomy and relevant safety issues. Korean J Anesthesiol.

[14] Bajwa SJ, Kaur J (2013). Clinical profile of levobupivacaine in regional anesthesia: a systematic review. J Anaesthesiol Clin Pharmacol.

[15] Burlacu CL, Buggy DJ (2008). Update on local anesthetics: focus on levobupivacaine. Ther Clin Risk Manag.

[16] Khot PP, Desai SN, Bale SP, Aradhya BN (2023). Comparison of ultrasound-guided paravertebral block versus erector spinae plane block for postoperative analgesia after percutaneous nephrolithotomy - a randomised, double-blind, controlled study. Indian J Anaesth.

[17] Elewa AM, Faisal M, Sjöberg F, Abuelnaga ME (2022). Comparison between erector spinae plane block and paravertebral block regarding postoperative analgesic consumption following breast surgery: a randomized controlled study. BMC Anesthesiol.

[18] Bryniarski P, Paradysz A, Zyczkowski M, Kupilas A, Nowakowski K, Bogacki R (2012). A randomized controlled study to analyze the safety and efficacy of percutaneous nephrolithotripsy and retrograde intrarenal surgery in the management of renal stones more than 2 cm in diameter. J Endourol.

[19] Coveney E, Weltz CR, Greengrass R, Iglehart JD, Leight GS, Steele SM, Lyerly HK (1998). Use of paravertebral block anesthesia in the surgical management of breast cancer: experience in 156 cases. Ann Surg.

[20] Swisher MW, Wallace AM, Sztain JF (2020). Erector spinae plane versus paravertebral nerve blocks for postoperative analgesia after breast surgery: a randomized clinical trial. Reg Anesth Pain Med.

